# Predictive models of diabetes complications: protocol for a scoping review

**DOI:** 10.1186/s13643-020-01391-w

**Published:** 2020-06-08

**Authors:** Ruth Ndjaboue, Imen Farhat, Carol-Ann Ferlatte, Gérard Ngueta, Daniel Guay, Sasha Delorme, Noah Ivers, Baiju R. Shah, Sharon Straus, Catherine Yu, Holly O. Witteman

**Affiliations:** 1 VDepartment of Family and Emergency Medicine, Faculty of Medicine, Université Laval, Quebec, Canada; 2 Faculté de Médecine, Université Laval, 1050, Avenue de la médecine, Quebec City, Quebec G1V A06 Canada; 3 Département de médecine sociale et préventive, Faculté de Médecine, Université Laval, 1050, Avenue de la médecine, Quebec City, Quebec G1V A06 Canada; 4 Diabetes Action Canada, Montreal, Quebec Canada; 5 Diabetes Action Canada, Regina, Saskatwewan Canada; 6 Family Practice Health Centre, Women’s College Hospital, 77 Grenville Street, Toronto, Ontario M5S 1B3 Canada; 7 Sunnybrook Health Sciences Centre, 2075 Bayview Ave., Room G106, Toronto, Ontario M4N 3M5 Canada; 8 Department of Medicine, University of Toronto, 27 King’s College Circle, Toronto, Ontario M5S 1A1 Canada; 9 Division of Endocrinology & Metabolism, St. Michael’s Hospital, Toronto, Ontario M5B 1W8 Canada

**Keywords:** Diabetes mellitus, Review, Risk prediction, Predictive models, Complication, Diabetic complication

## Abstract

**Background:**

Diabetes is a highly prevalent chronic disease that places a large burden on individuals and health care systems. Models predicting the risk (also called predictive models) of other conditions often compare people with and without diabetes, which is of little to no relevance for people already living with diabetes (called patients). This review aims to identify and synthesize findings from existing predictive models of physical and mental health diabetes-related conditions.

**Methods:**

We will use the scoping review frameworks developed by the Joanna Briggs Institute and Levac and colleagues. We will perform a comprehensive search for studies from Ovid MEDLINE and Embase databases. Studies involving patients with prediabetes and all types of diabetes will be considered, regardless of age and gender. We will limit the search to studies published between 2000 and 2018. There will be no restriction of studies based on country or publication language. Abstracts, full-text screening, and data extraction will be done independently by two individuals. Data abstraction will be conducted using a standard methodology. We will undertake a narrative synthesis of findings while considering the quality of the selected models according to validated and well-recognized tools and reporting standards.

**Discussion:**

Predictive models are increasingly being recommended for risk assessment in treatment decision-making and clinical guidelines. This scoping review will provide an overview of existing predictive models of diabetes complications and how to apply them. By presenting people at higher risk of specific complications, this overview may help to enhance shared decision-making and preventive strategies concerning diabetes complications. Our anticipated limitation is potentially missing models because we will not search grey literature.

## Background

The World Health Organization identifies diabetes as one of the four priority non-communicable conditions [1]. In 2017, more than 693 million people were affected by diabetes worldwide and projections point to a sustained rise in its prevalence in the next decades [1]. The burden of diabetes on individuals and health care systems is primarily attributed to complications from diabetes including macrovascular complications (e.g., heart attack, stroke) or microvascular complications (e.g., blindness, amputation, renal failure) [1, 2]. Early identification of people with diabetes at increased risk of complications is an important challenge for clinicians [3]. Models predicting the risk (also called predictive models) of diabetes complications can facilitate the identification of people at higher risk and inform health decision-making regarding preventive actions or treatments to avoid or delay complications [4].

Models that assess the risk of developing diabetes or that use it as a predictor variable for other outcomes are not informative for someone who is already living with diabetes (i.e., patient) [5, 6]. Similarly, predictive models of other conditions in people with diabetes often compare people with and without diabetes, which is of little to no relevance for patients [7–9]. A preliminary search for reviews on the topic was conducted in two databases (MEDLINE, Embase), and results suggest that existing reviews of predictive models of diabetes-related complications focus mostly on macrovascular complications [10, 11] and rarely on the range of other diabetes complications [4, 12]. This scoping review will contribute to filling these gaps.

We aim to identify and synthesize existing predictive models of physical and mental health conditions associated with diabetes, in people with prediabetes and any type of diabetes mellitus (hereafter called “patients”). Our objective is to describe the features of selected validated predictive models for risk of diabetes complications.

## Methods/design

In this scoping review, we will use well-established scoping review methods, namely the framework developed by the Joanna Briggs Institute [13, 14] and Levac and colleagues [15] while paying attention to the methodological limitations of original studies as often recommended in systematic reviews [16]. In some epidemiological contexts, such as the one we are focusing on, it is important to assess studies’ qualities even if it does not add to the methodological strength of the scoping review itself. For example, in an ongoing scoping review, authors aimed to assess the number of validated prediction rules that exist for spinal cord injury management and to provide evidence of the psychometric properties of these prediction rules, especially with regard to its clinical impact [17]. Although their scoping approach does not aim to assess the overall effectiveness of these prediction rules in their respective settings, their systematic appraisal of data quality will help readers make informed use of their findings. In another ongoing study, authors aimed to “produce a scoping review which in its data analysis will draw on methods typically associated with qualitative systematic reviews” and acknowledged that the diversity of data “presents a potential challenge from the perspective of interrater reliability and consistency in analysis” [18].

To include a diversity of perspectives and ensure that our review focuses on diabetes complications that are relevant to patients [19], our research team include researchers (RN, IF, GN, HW) and stakeholders such as clinicians (CF, BS, CY, NI, SS) and patients with type 1 and type 2 diabetes (DG, DA, HW). Stakeholders were involved in this study as collaborators and co-authors, not participants. Patients in our research team (hereafter called Expert Patients) were recruited through Diabetes Action Canada (DAC), a national Patient-Oriented Research Network that includes patients to bring expertise in diabetes care [20]. Expert Patients were recruited to DAC through professional and personal networks and community-based organizations and from respondents to a national survey [21]. Using a patient-centered approach, the team co-developed the protocol. We integrated patient’ priorities by developing our research questions, search strategy terms, and outcome measures based on what Expert Patients shared concerning what matters to them, and also by building on findings of a recent patient-centered study [21]. Expert Patients (DG, SD) will be involved in each step of the research process, including the definition of the objective, the main analysis, the preliminary and final results, and the discussion. We will discuss preliminary and final results with a broader committee of six to ten Expert Patients. We will use the services of two information specialists to validate our search strategy and selection criteria at least twice before the end of this review.

### Eligibility criteria

#### Population

The population targeted by this scoping review consists of people of all ages, genders, and ethnicities affected by diabetes. We will consider prediabetes and  any type of diabetes, including type 1, and type 2 diabetes [22], and data that have been collected at the individual level, not the group level [23]. We will consider both treated and non-treated individuals. Studies mixing people with and without diabetes will not be considered, unless they performed separate stratified analyses for individuals with diabetes and without diabetes. Studies of pregnant women and/or gestational diabetes will be excluded because it is a different clinical condition. Studies that are restricted to people who do not have diabetes will not be considered. Models based on the Framingham Risk Score of cardiovascular conditions will not be considered as this score was originally derived from a general population free of diabetes [24]. Studies involving people not meeting our eligibility criteria will be excluded.

#### Concept

We will consider both clinically diagnosed and self-reported physical and mental conditions experienced by patients as a consequence of living with diabetes. Studies focusing on social or economic consequences of diabetes will not be included in this review, because findings are likely to be highly dependent on country of residence and health insurance status and thus are unlikely to be modifiable at the individual level. We plan to sort models by diabetes type and by groups (e.g., sub-group) of diabetes complications, physical (e.g., macrovascular and microvascular conditions), and mental (e.g., depression and anxiety) health problems. Death from all causes and death from non-diabetes complications will be analyzed separately. With the collaboration of Expert Patients and researchers, we drafted a preliminary and non-exhaustive list of diabetes complications that were relevant for patients (Table 1).

**Table 1:** List of diabetes complications for inclusion in the search strategy

Categories	Specific complications
Cardiovascular and coronary diseases	Heart failure/heart attack/myocardial infarction Stroke Chest pain/angina/coronary syndrome Atherosclerosis
Kidney damage and other nephropathy	Chronic kidney disease/renal disease Kidney failure/irreversible renal insufficiency Urinary tract infection
Eye damage	Diabetic retinopathy Macular edema/cataracts/glaucoma Vision loss/blindness/vision impairment
Nerve damage	Diabetic peripheral neuropathy Erectile dysfunction/hypogonadism Foot damage/diabetic foot/amputation Infections/ulcers Ischemia
Musculoskeletal complications	Diabetic arthropathy/neuropathic arthropathy Charcot’s joint
Oral complications	Periodontitis
Respiratory complications	Obstructive sleep apnea
Mental health complications	Depression/anxiety/diabetes distress
Acute complications	Ketoacidosis/hyperosmolar hyperglycemic state Hypoglycemia/hyperglycemic diabetic coma Fainting
Others	Death/mortality

#### Context

(1) We will consider evidence coming from all countries and settings and published between 2000 and 2018. We will not consider articles prior to 2000 because both diabetes treatment and modeling approaches have greatly improved in the last two decades. The date of publication will not be included in the search strategy. Rather, we will simply order the results by date of publication and will not consider those outside the period 2000–2018. (2) We will include only full-text peer-reviewed published studies with original results as they are expected to exhibit high-quality models and detailed methodology. For this reason, we will not consider abstracts only or duplicates and do not intend to search the grey literature. (3) No language restrictions will be applied. During the full-text screening, potentially relevant articles written in a language other than English or French will be translated by a member of our team when possible. If we do not have anyone with expertise in that language, we will first use free translation tools (e.g., Google Translate, DeepL) to determine if the publication is likely to meet our inclusion criteria, and if so, we will engage professional translation services. (4) We will only consider studies with a longitudinal design and quantitative data. Specifically, we will consider prospective cohort studies and nested case-control studies [25]. We will not apply restrictions as to the length of follow-up as the time may vary for diverse reasons. Screening tools/studies, retrospective case-control studies, and cross-sectional studies will not be considered. Focusing on predictive models implies that we will not consider explicative ones, that is, those evaluating factors associated with diabetes complications as potential determinants or confounders rather than predictors. We will consider diverse candidate/potential predictors of diabetes complications, including personal characteristics, socioeconomic factors, clinical factors, and environmental factors. (5) We will focus only on prognostic models and not include diagnostic models in this review. We will consider both development and validation studies, as some studies presenting predictive models are focused on derivation and internal validation and others on external validation. The sample size for model validation can come from the same study population, from another study population, or from both. We will exclude partial and full predictive models that were not validated, either internally or externally.

### Search strategy and information source

Our diverse team co-built the search strategy of this scoping review. A predefined list of potential predictors and complications [4] was established in collaboration with six Expert Patients who were not members of our research team in order to better capture what matters to diverse patients. This list will be used as a starting point for study selection and will be revised during the full-text screening process (Table 2). The search strategy will combine groups of keywords customized to each database (i.e., MeSH terms where appropriate) pertaining to (1) population (treated and untreated patients affected by prediabetes and diabetes), (2) concept (diabetes complications, potential predictors), and (3) context (prediction modeling features). Prediction models seldom report the individual predictors included in the final model as the central message is about accuracy (discrimination and calibration). However, knowing which, how, and what candidate predictors have been assessed can help explore potential bias (e.g., selection bias) in data that may, in turn, influence the features of predictive models [26, 27]. For this reason, we will add potential predictors in our search strategy. Search terms are selected to capture international terminology. We intend to run a search at the start and again just before final data extraction to identify studies published after our baseline search date and before we write the article for possible inclusion in our review. As mentioned in eligibility criteria, there will be no restrictions in terms of date, language, age, or design.

**Table 2:** Non-exhaustive list of potential predictors of diabetes complications

Categories	Predictors
Individual characteristics	Age Gender/sex Ethnicity/race/language/culture Place of birth Geography/residence characteristics Education Socioeconomic status/household income/unemployment Marital status
Lifestyle-related factors	Physical activity/inactivity Smoking/illicit drugs Alcohol consumption Eating/food habits/unhealthy diet
Psychosocial factors	Stress Social deprivation/loneliness Social factors/psychosocial constraints
Clinical characteristics	Family history Lipids Blood pressure BMI/obesity/waist to hip ratio/weight Presence/duration of diabetes/age of first diabetes diagnosis Glycemic control/glycated hemoglobin/self-care Medication adherence/treatment/medication

We will search for eligible studies in two electronic scientific databases: Ovid MEDLINE and Embase. In addition, we will perform snowballing of reference lists of selected papers at the full-text screening stage [28]. To complement these sources, we will contact experts in the field to ask if they know about any published work we may have missed. We tested our search strategy for MEDLINE (Ovid) in June 2018 and for Embase in October 2018 (see Appendixes 1, 2, and 3). We had the search appraised by a second librarian using PRESS in October 2018 [29].

#### Data management

The detailed references and abstracts identified will be pooled in EndNote, a reference management software [30]. We will use EndNote to remove duplicates and store references before moving to another tool to screen references and extract data. Duplicates will be removed using the automatic function in EndNote and manually during screening. Screening by title, abstract, and full text will be conducted using Microsoft Excel [31] to provide a comprehensive step-by-step record of the selection process based on our selection criteria. A detailed screening form with the inclusion and exclusion criteria will be developed and tested (see Appendixes 1 and 2, Tables 4 and 5). All members of the screening team will be trained on how to use Microsoft Excel and the screening form before we start.

#### Selection process

Articles will be excluded if at least one of the criteria was clearly not met. We will retain any article that cannot be excluded solely based on abstract review. We will set aside all articles that are systematic or narrative literature reviews whose subject clearly relates to our objective to consult at a later stage, as mentioned previously.

Given that reviewers have diverse research backgrounds and levels of experience, we plan to screen titles and abstracts in two different steps to make sure that they have a similar understanding of the eligibility criteria. A preliminary convenience sample of 50 titles will be screened by all reviewers, and we will assess the degree of agreement among raters, discuss any disagreement in groups, and only proceed above a predetermined threshold of interrater agreement (such as 70%). Then, pairs of reviewers from among the seven team members (CF, IF, JC, SC, SRB, JM, YY) will independently screen a subset of titles based on the Population-Concept-Context (PCC) criteria. After titles are screened independently by two reviewers, the results will be pooled and agreement will be calculated for each pair. If agreement is optimal, all titles retained by at least one reviewer will be considered for abstract screening. If agreement is not optimal, title screening will be repeated by independent reviewers until we meet the target of 0.7 or higher. Reviewers will meet at the beginning, midpoint, and final stages of the abstract review process to discuss discrepancies related to study selection and refine the search strategy if needed [15]. Once abstract screening has been completed by two independent reviewers, the results will be pooled and agreement will be calculated for each pair of reviewers. When agreement is optimal, all remaining disagreements will be discussed between the two reviewers. If agreement is not optimal, two independent reviewers will screen abstracts until we meet the target agreement of 0.7 or higher. A third reviewer will screen abstracts where there are discrepancies and discuss all remaining disagreements in meetings with the two initial reviewers. Full-text copies of articles selected based on abstracts will be retrieved and translated if needed. Two independent reviewers from our team (RN, CRB, TP) will screen the full text of all selected references. Each pair of reviewers will compare their results and discuss any disagreement. If there are too many disagreements, a third reviewer will repeat the full-text screening. Differences and disagreements between reviewers will be discussed in group meetings to reach a consensus. All remaining discrepancies will be resolved by one researcher (GN, HW).

#### Data collection process

The team will collectively build a standardized extraction grid with all relevant data items to guide data extraction. Three independent reviewers (RN, TP, CRB) will pilot test the grid using a subset of five to twenty full-text articles selected for extraction. They will then meet to determine whether data are missing from the form or not needed. Data extraction will be performed in duplicate by two independent reviewers from our team (RN, TP, CRB). The corresponding authors of retained articles may be contacted to request any information missing in the extraction grid. The three reviewers will resolve discrepancies through discussion and with input from two members of our team (RN, HW) when necessary.

### Data extraction

Since there are no checklists of items to consider in data extraction for scoping reviews on risk prediction models, we considered aspects of a well-known checklist for systematic reviews [32] that aligns with the scoping review methodology to design (and, in future, report) our data extraction process [15]. Full-text data extraction will be done by two independent reviewers (TP, CRB) using an Excel spreadsheet. A third reviewer (RN, GN) will review any studies where there is a discrepancy between the two independent reviewers that they are not able to resolve. Although scoping reviews do not usually include quality assessment, when dealing with epidemiological models, it is important to pay attention to the methodology and the design of original studies [17]. Two independent reviewers trained in epidemiology (RN, IF, GN) will be involved in assessing potential selection and information bias in selected studies and will discuss the potential impact of bias on the features and accuracy of selected models. Final selection of articles will be undertaken in duplicate following data extraction to confirm relevance of the chosen articles. Any study selected by only one reviewer will be discussed to reach mutual agreement. We will record the reasons for which each article is excluded. Here again, a third reviewer will review each study when there are discrepancies that cannot be resolved by the two independent reviewers.

We will use the pre-publication version of the PROBAST [33], which includes a template and a detailed user guide to identify five domains in which methodological limitations might exist in studies using risk prediction models. These domains are as follows: (1) participant selection (e.g., selection bias caused by exclusion of eligible participants or loss at follow-up); (2) predictors (e.g., differential or non-differential misclassification of predictors, change in predictor for some participants over time); (3) outcomes (e.g., outcome definition and standardized classification of all participants); (4) sample size and participation flow (e.g., inappropriate time interval between predictor and outcome measurements, handling of missing data); and (5) analyses (e.g., evaluation of performance measures such as calibration, discrimination, (re)classification, and net benefit [34–36]; handling of non-binary predictors) (Table 3). Other methodological issues will also be considered (e.g., duration and timing of exposure, selective reporting of results in a way that depends on the findings) [37]. Also, if both predictors and outcomes were measured using self-report methods, we will evaluate potential common method bias [25].We will use the same spreadsheet for data abstraction and for quality assessment. We will make sure that we adequately capture all relevant content and methods from selected papers and summarize information on the internal and external validity of each selected model from each selected study. Consistent with the PROBAST tool, we will sort studies in three groups: high quality, moderate/acceptable quality, and low quality. These data will help assess data quality during data analysis and interpretation.

**Table 3:** Data to extract from selected eligible full text

	Description	Items
1.	Derivation and validation population	Year of study, country, sample size, date of recruitment, participation and attrition rates, mean age, gender, socioeconomic status, etc.
2.	Study design	Prospective, retrospective, case-cohort, duration of follow-up
3.	Predictors	Source of data, definitions, and measurement methods, variable categorization, time(s) predictors were measured, variation in time
4.	Outcome events	Prevalence, source of data, definition, measurement, blinded assessment or not
5.	Analysis	Prognostic prediction model, modeling method, list and selection of predictors candidates, treatment of missing data and losses at follow-up, sensitivity analyses, stratified analyses, interaction tests, model performance, etc.
6.	Results	Name of each outcome, frequency estimates of outcomes, estimates with confidence intervals or *p* values for each prediction model by predictors and by diabetes-related complications, alternative presentation of the models
7.	Potential limitations	Selection bias (percentage participation at baseline and at follow-up, missing data), information bias (measurement of exposure and/or outcome), lack of power, statistics of the performance of the model (validation, calibration, discrimination
8.	Interpretation	Utility of presented models, generalization of the findings

### Analysis and synthesis

This protocol adheres to the Preferred Reporting Items in Systematic Reviews and Meta-analyses extension for protocols (PRISMA-P) [38] and scoping reviews (PRISMA-ScR) [39] (see the Additional file 1). After data from included studies are summarized in an extraction table, we will follow three distinct steps: analysis (models features, discrimination, calibration and validation), reporting (synthesizing characteristics of included studies), and discussion (comparison with previous reviews) [15, 40]. The analysis and synthesis will focus on diabetes complications and the methodological features of selected models [11]. We will use qualitative approaches to evaluate and synthesize quantitative estimates accurately. When relevant, we will provide in-depth analyses of potential explanations for data inconsistencies (i.e., study design, selection/participation, data measurements, etc.). Finally, we will propose how to consistently report the risk of diabetes complications in predictive models in ways that will be helpful for patients and clinicians.

## Discussion and conclusion

The current review may not provide meta-analytical estimates because we expect to retrieve a highly diverse set of risk prediction models. This may preclude a quantitative synthesis if the available data do not meet the criteria for homogeneity in methods used to measure predictors and outcomes and assess biases potentially affecting internal validity. Heterogeneity is one of the main reasons for skepticism about meta-analyses of non-experimental studies [25, 41], which represent the great majority of studies on our topic [4, 6]. To partly circumvent the pitfalls of heterogeneity, we will attempt to calculate a meta-analytical estimate of experimental studies if there are enough high-quality data with comparable methodological characteristics in our final set of models (*N* > 5). However, preliminary search results and consultation with experts revealed that predictive models of diabetes complications often consider some complications as predictors of other complications [4]. Merging such models during analysis may lead to a highly correlated data and inflation in the estimates of variance [42, 43]. In such cases, qualitative approaches are often alternatives used to evaluate and synthesize estimates accurately.

### Strengths and limitations of this study

The major strengths of this review will be the inclusion of predictive models of diverse diabetes complications and the combination of multiple and diverse perspectives of patients, clinicians, and researchers. Considering the fact that diabetes complications often vary by diabetes types, we invited one patient partner with type 2 diabetes (DG) and one patient partner with type 1 diabetes (SD) as co-authors to complement the perspective of our senior researcher (HW) who lives with type 1 diabetes. All six Expert Partners that we consulted agreed that all complications considered in this review were equally important. We plan to actively collaborate with a committee of Expert Patients, caregivers, and clinicians in diabetes care. By including a consultation exercise in this scoping review, we intend to “enhance the results, making them more useful to policy makers, practitioners and service users” [44]. Limitations include using two databases, restricting publication date to 2000–2018, and not searching the grey literature. Also, we will not consider the social and economic outcomes of diabetes.

### Dissemination

Ethical approval is not required for this scoping review study since we will only be using secondary data sources. Our findings will be disseminated through peer-reviewed publication and presentation at conferences. Because predictive models are increasingly being appraised and recommended for formal risk assessment in treatment decision-making and clinical guidelines, the proposed scoping review may contribute to support research and risk communication in diabetes care. For example, it may help clinicians better identify people who are at higher risk of diabetes complications and researchers design customizable risk prediction tools for use in diabetes care [45]. To ensure that our findings about diabetes complications reach patients, we will also circulate them through clinical and patient networks.

## Supplementary information

**Additional file 1.** PRISMA-P 2015 checklist.

## Data Availability

Data are available by requesting to the corresponding author.

## Appendix 1

**Table 4:** Ovid research strategies (submitted on April 2020). Database(s): Ovid MEDLINE(R) ALL 1946 to April 15, 2020. Search strategy

#	Searches	Results
1	Prediabetic State/co or Diabetes Mellitus/ or Diabetes Mellitus, Type 1/co or exp Diabetes Mellitus, Type 2/co or HYPERGLYCEMIA/co	164363
2	(Prediabetic or Prediabetes).ti,ab.	7180
3	Diabetes.ti,ab.	507372
4	Hyperglycemia?.ti,ab.	41791
5	(Hyperglycemic adj2 (States or Syndrome)).ti,ab.	286
6	insulin resistance/ or metabolic syndrome/	81615
7	VALIDATION STUDIES AS TOPIC/ or VALIDATION STUDIES/	102046
8	(predictive adj2 (accuracy or equation or model or rule or tool or value)).ti,ab.	109765
9	(risk adj2 (calculator or model)).ti,ab.	11269
10	(prediction adj2 (model or rule or tool)).ti,ab.	16401
11	early prediction.ab,ti.	2634
12	area under curve/ or linear models/ or logistic models/ or proportional hazards models/ or roc curve/ or survival analysis/ or disease-free survival/ or kaplan-meier estimate/	565422
13	“Predictive Value of Tests”/	200163
14	age factors/ or comorbidity/ or sex factors/	689245
15	“emigrants and immigrants”/ or undocumented immigrants/ or population groups/ or continental population groups/ or african continental ancestry group/ or african americans/ or american native continental ancestry group/ or alaska natives/ or indians, central american/ or indians, north american/ or indians, south american/ or inuits/ or asian continental ancestry group/ or asian americans/ or european continental ancestry group/ or oceanic ancestry group/ or ethnic groups/ or amish/ or arabs/ or roma/ or hispanic americans/ or mexican americans/ or jews/ or “geographicals (non mesh)”/ or geographic locations/	302552
16	Socioeconomic Factors/	154236
17	INCOME/	28804
18	family characteristics/ or marital status/	33832
19	educational status/ or academic failure/ or literacy/	51152
20	education.ab,ti.	434536
21	((Socioeconomic or Income? or salar* or Racial or race) adj6 (disparit* or characteristic? or Inequalit* or factor? or distribution)).ti,ab.	48938
22	Residence Characteristics/	33032
23	(“Residence Characteristic?” or “place of birth” or Neighborhood? or “Birth Place” or Communit*).ab,ti.	568981
24	Medical History Taking/	19270
25	(Family adj2 histor*).ab,ti.	61729
26	Exercise/	107145
27	Sedentary Lifestyle/	8997
28	(Sedentary or “Physical inactivity” or “Physical Activity”).ab,ti.	126856
29	smoking/ or tobacco smoking/ or cigarette smoking/ or “tobacco use”/	142398
30	smoking.ab,ti.	217829
31	Alcohol Drinking/	66190
32	(Alcohol adj2 (drinking or consumption)).ti,ab.	51153
33	DIET/ or “DIET, FOOD, AND NUTRITION”/ or DIET THERAPY/	166383
34	Feeding Behavior/	81254
35	((Diet* or Food or Eat*) adj3 (Habit? or Pattern? or Behavior? or unhealthy)).ti,ab.	48072
36	“body weights and measures”/ or body fat distribution/ or body mass index/ or body size/ or waist circumference/ or waist-height ratio/ or waist-hip ratio/	151455
37	OBESITY/	177706
38	(Obesity or Overweight or BMI or Weight).ab,ti.	1042711
39	(Waist adj2 “Hip Ratio”).ab,ti.	9404
40	Social Class/ or Social Isolation/	52910
41	LONELINESS/	3573
42	(“Social Deprivation” or loneliness).ab,ti.	7478
43	Glycated Hemoglobin A/	34396
44	(“duration of diabetes” or “glycemic control”).ab,ti.	31221
45	MEDICATION ADHERENCE/	18509
46	GLUCOCORTICOIDS/	63253
47	Glucocorticoid?.ab,ti.	66928
48	exp diabetes complications/ or exp diabetic angiopathies/ or exp diabetic foot/ or exp diabetic retinopathy/ or exp diabetic cardiomyopathies/ or exp diabetic coma/ or exp hyperglycemic hyperosmolar nonketotic coma/ or exp diabetic ketoacidosis/ or exp diabetic nephropathies/ or exp diabetic neuropathies/ or exp fetal macrosomia/	130241
49	Mortality/	43499
50	(Mortality or mortalities or “death rate”).ab,ti.	751942
51	hypoglycemia/ or insulin coma/	27599
52	cardiovascular diseases/ or heart diseases/ or heart arrest/ or exp death, sudden, cardiac/ or out-of-hospital cardiac arrest/ or exp heart failure/ or myocardial ischemia/ or exp acute coronary syndrome/ or exp angina pectoris/ or exp coronary disease/ or exp myocardial infarction/	750013
53	STROKE/ or HEAT STROKE/	100473
54	ATHEROSCLEROSIS/	35467
55	Hypertension/	232193
56	CHOLESTEROL/	119027
57	Dyslipidemias/	11293
58	(heart adj2 (disease or failure or attack or Defect* or Arrest or Rupture)).ab,ti.	316980
59	Hypoglyc?emia.ab,ti.	38892
60	“Angiopath*”.ab,ti.	5883
61	High Blood Pressure?.ab,ti.	14717
62	stroke?.ab,ti.	241610
63	Angina.ab,ti.	52094
64	“Atheroscleros*”.ab,ti.	110940
65	Hypertension.ab,ti.	373532
66	“Nephropath*”.ab,ti.	56336
67	kidney diseases/ or diabetes insipidus/ or diabetic nephropathies/ or exp renal insufficiency/ or urinary tract infections/	309073
68	Kidney Failure, Chronic/ or Renal Insufficiency, Chronic/	113218
69	Macular Edema/	6942
70	Blindness/	19770
71	GLAUCOMA/	35861
72	BLINDNESS/	19770
73	CATARACT/	28378
74	((Kidney or Renal) adj3 (Insufficienc* or Disease? or Failure? or problem? or complication?)).ab,ti.	241533
75	Urinary Tract Infection?.ab,ti.	39613
76	Macular Edema.ab,ti.	9536
77	((Visual or Vision or eye) adj2 (Disorder? or Impairment? or loss or complication?)).ab,ti.	43813
78	Cataract.ab,ti.	47352
79	Glaucoma?.ab,ti.	56406
80	(nerve adj2 (damage or complication)).ab,ti.	6744
81	Erectile Dysfunction.ab,ti.	15344
82	HYPOGONADISM/	8605
83	ISCHEMIA/	49573
84	exp Diabetic Foot/ or Foot Ulcer/ or exp Diabetes Complications/ or exp Diabetic Neuropathies/	131425
85	joint diseases/ or arthropathy, neurogenic/	26138
86	Arthropathy, Neurogenic/	1693
87	ARTHRITIS/	35382
88	OSTEOARTHRITIS/	36682
89	((Foot or Plantar) adj2 Ulcer*).ab,ti.	6420
90	((Foot or leg or toe) adj2 damage).ab,ti.	81
91	“Charcot’s joint”.ab,ti.	58
92	Ischemia.ab,ti.	174311
93	Hyperglycemia?.ab,ti.	41791
94	HYPOGLYCEMIA/	27104
95	PERIODONTITIS/	17789
96	Anxiety/	79355
97	DEPRESSION/	116489
98	Mental Health/	37148
99	“Quality of Life”/	190705
100	(“diabetes distress” or “diabetes burden”).ab,ti.	631
101	Sleep Apnea, Obstructive/	19646
102	PERIODONTITIS/	17789
103	Patient Reported Outcome Measures/	5360
104	“patient-reported experience measure*”.ab,ti.	108
105	Stress, Psychological/	118727
106	(risk adj2 (calculator or model)).ti,ab.	11269
107	1 or 2 or 3 or 4 or 5 or 6	619369
108	7 or 8 or 9 or 10 or 11 or 106	231925
109	7 or 8 or 9 or 10 or 11 or 12 or 13	898232
110	14 or 15 or 16 or 17 or 18 or 19 or 20 or 21 or 22 or 23 or 24 or 25 or 26 or 27 or 28 or 29 or 30 or 31 or 32 or 33 or 34 or 35 or 36 or 37 or 38 or 39 or 40 or 41 or 42 or 43 or 44 or 45 or 46 or 47	3613933
111	14 or 15 or 16 or 17 or 18 or 19 or 20 or 21 or 22 or 23 or 24 or 25 or 26 or 27 or 28 or 29 or 30 or 31 or 32 or 33 or 34 or 35 or 36 or 37 or 38 or 39 or 40 or 41 or 42 or 43 or 44 or 45 or 46 or 47 or 106	3621708
112	48 or 49 or 50 or 51 or 52 or 53 or 54 or 55 or 56 or 57 or 58 or 59 or 60 or 61 or 62 or 63 or 64 or 65 or 66 or 67 or 68 or 69 or 70 or 71 or 72 or 73 or 74 or 75 or 76 or 77 or 78 or 79 or 80 or 81 or 82 or 83 or 84 or 85 or 86 or 87 or 88 or 89 or 90 or 91 or 92 or 93 or 94 or 95 or 96 or 97 or 98 or 99 or 100 or 101 or 102 or 103 or 104 or 105	3543083
113	107 and 108 and 111 and 112	2629
114	107 and 109 and 111 and 112	18759
115	113 OR 114	18759

## Appendix 2

**Table 5:** Embase research strategies (submitted on April 2020). Database(s): Embase.com 1946 to April 15, 2020

#	Searches	Results
1	“impaired glucose tolerance”/de/dm_co OR “diabetes mellitus”/de OR “insulin dependent diabetes mellitus”/exp/dm_co OR “non insulin dependent diabetes mellitus”/exp/dm_co OR “hyperglycemia”/dm_co	574821
2	(Prediabetic or Prediabetes):ti,ab	11113
3	Diabetes:ti,ab	781146
4	Hyperglycemia*:ti,ab	62514
5	(Hyperglycemic NEAR/2 (States or Syndrome)):ti,ab	368
6	“insulin resistance”/de OR “metabolic syndrome X”/de	183355
7	“validation study”/de	82278
8	(predictive NEAR/2 (accuracy or equation or model or rule or tool or value)):ti,ab	164979
9	(risk NEAR/2 (calculator or model)):ti,ab	17522
10	(prediction NEAR/2 (model or rule or tool)):ti,ab	23950
11	“early prediction”:ab,ti	4040
12	“area under the curve”/exp OR “statistical model”/de OR “proportional hazards model”/de OR “receiver operating characteristic”/de OR “survival analysis”/de OR “disease free survival”/de OR “Kaplan Meier method”/de	609166
13	“predictive value”/de	166618
14	“age”/de OR “comorbidity”/de OR “sex factor”/de	776824
15	“migrant”/de OR “emigrant”/de OR “immigrant”/de OR “undocumented immigrant”/de OR “population group”/de OR “ancestry group”/de OR “Black person”/de OR “African American”/de OR “Asian American”/de OR “Asian continental ancestry group”/de OR “indigenous people”/exp OR “Inuit”/de OR “Caucasian”/de OR “Oceanic ancestry group”/exp OR “ethnic group”/de OR “Amish”/de OR “Arab”/de OR “Romani (people)” /de OR “Hispanic”/exp OR “Jew”/exp	379246
16	“socioeconomics”/de	144345
17	“income”/de	60594
18	“household income”/exp OR “family size”/de OR “marriage”/de	84364
19	“educational status”/exp OR “academic failure”/de	76673
20	education:ab,ti	586461
21	((Socioeconomic or Income* or salar* or Racial or race) NEAR/6 (disparit* or characteristic* or Inequalit* or factor* or distribution)):ti,ab	64079
22	(“Residence Characteristic*” or “place of birth” or Neighborhood* or “Birth Place” or Communit*):ab,ti	702190
23	“anamnesis”/exp	223851
24	(Family NEAR/2 histor*):ab,ti	106324
25	“exercise”/de	282979
26	“sedentary lifestyle”/de	13705
27	(Sedentary or “Physical inactivity” or “Physical Activity”):ab,ti	171444
28	“smoking”/exp OR “tobacco use”/exp	395742
29	smoking:ab,ti	315649
30	“drinking behavior”/de	49702
31	(Alcohol NEAR/2 (drinking or consumption)):ti,ab	70214
32	“diet”/de OR “nutrition”/de OR “diet therapy”/de	378305
33	“feeding behavior”/de OR “eating habit”/de	96395
34	((Diet* or Food or Eat*) NEAR/3 (Habit* or Pattern* or Behavior* or unhealthy)):ti,ab	68633
35	“morphometry”/de OR “body fat distribution”/de OR “body mass”/de OR “abdominal circumference”/de OR “body fat”/de OR “body size”/de OR “fat mass”/de OR “waist circumference”/de OR “waist to height ratio”/de OR “waist hip ratio”/de	552417
36	“obesity”/de	423629
37	(Obesity or Overweight or BMI or Weight):ab,ti	1482934
38	(Waist NEAR/2 “Hip Ratio”):ab,ti	14075
39	“social class”/de OR “social isolation”/de	55517
40	“loneliness”/de	7849
41	(“Social Deprivation” or loneliness):ab,ti	9541
42	“glycosylated hemoglobin”/exp	123256
43	(“duration of diabetes” or “glycemic control”):ab,ti	50242
44	“medication compliance”/de	30458
45	“glucocorticoid”/de	86402
46	Glucocorticoid*:ab,ti	87882
47	“diabetic complication”/exp OR “diabetic angiopathy”/exp OR “diabetic foot”/de OR “diabetic retinopathy”/exp OR “diabetic cardiomyopathy”/de OR “diabetic coma”/exp OR “macrosomia”/de	153567
48	“mortality”/de	758705
49	(Mortality or mortalities or “death rate”):ab,ti	1102207
50	“hypoglycemia”/de OR “hypoglycemic coma”/de	80964
51	“cardiovascular disease”/de OR “heart disease”/de OR “heart arrest”/de OR “sudden cardiac death”/de OR “occupational sudden death”/de OR “out of hospital cardiac arrest”/de OR “heart failure”/de OR “acute coronary syndrome”/de OR “angina pectoris”/exp OR “coronary artery disease”/exp OR “heart muscle ischemia”/de OR “heart infarction”/de OR “anterior myocardial infarction”/de OR “inferior myocardial infarction”/de OR “non ST segment elevation myocardial infarction”/de OR “ST segment elevation myocardial infarction”/de OR “anterior myocardial infarction”/de OR “cardiogenic shock”/de OR “systolic dysfunction”/de OR “diastolic dysfunction”/de OR “cardiorenal syndrome”/de OR “cardiorenal syndrome”/de OR “paroxysmal dyspnea”/de OR “heart edema”/de	1266385
52	“stroke patient”/de OR “heat stroke”/de	34539
53	“atherosclerosis”/de	145020
54	“hypertension”/de	595997
55	“cholesterol”/de	206512
56	“dyslipidemia”/de	69924
57	(heart NEAR/2 (disease or failure or attack or Defect* or Arrest or Rupture)):ab,ti	487830
58	Hypoglyc?emia:ab,ti	17132
59	“Angiopath*”:ab,ti	8651
60	“High Blood Pressure*”:ab,ti	21975
61	stroke*:ab,ti	385606
62	Angina:ab,ti	75209
63	“Atheroscleros*”:ab,ti	155894
64	Hypertension:ab,ti	579851
65	“Nephropath*”:ab,ti	77558
66	“kidney disease”/de OR “diabetes insipidus”/exp OR “kidney failure”/exp OR “urinary tract infection”/de	618220
67	“macular edema”/exp	20227
68	“blindness”/de	37380
69	“glaucoma”/de	61605
70	“cataract”/de	53559
71	((Kidney or Renal) NEAR/3 (Insufficienc* or Disease* or Failure* or problem* or complication*)):ab,ti	353433
72	“Urinary Tract Infection*”:ab,ti	60232
73	“Macular Edema”:ab,ti	13366
74	((Visual or Vision or eye) NEAR/2 (Disorder* or Impairment* or loss or complication*)):ab,ti	59042
75	Cataract:ab,ti	58666
76	Glaucoma*:ab,ti	74138
77	(nerve NEAR/2 (damage or complication)):ab,ti	9310
78	“Erectile Dysfunction”:ab,ti	24704
79	“hypogonadism”/de	16719
80	“ischemia”/de	82529
81	“diabetic foot”/de OR “foot ulcer”/de OR “diabetic complication”/exp OR “diabetic neuropathy”/de	149679
82	“neuropathic joint disease”/de OR “arthropathy”/de	30639
83	“arthritis”/de	74468
84	“osteoarthritis”/de	86054
85	((Foot or Plantar) NEAR/2 Ulcer*):ab,ti	8899
86	((Foot or leg or toe) NEAR/2 damage):ab,ti	114
87	“Charcot* joint”:ab,ti	185
88	Ischemia:ab,ti	244068
89	Hyperglycemia*:ab,ti	62514
90	“hypoglycemia”/de	80458
91	“periodontitis”/de	25830
92	Anxiety/de	206353
93	“depression”/de	368849
94	“mental health”/de	137639
95	“quality of life”/de	457545
96	(“diabetes distress” or “diabetes burden”):ab,ti	1072
97	“sleep disordered breathing”/de	75851
98	“periodontitis”/de	25830
99	“patient-reported outcome”/de	21228
100	“patient-reported experience measure*”:ab,ti	217
101	“mental stress”/de	82976
102	(risk NEAR/2 (calculator or model)):ti,ab	17522
103	#1 OR #2 OR #3 OR #4 OR #5 OR #6	1 089 628
104	#7 OR #8 OR #9 OR #10 OR #11 OR #102	280 005
105	#7 OR #8 OR #9 OR #10 OR #11 OR #12 OR #13	917 540
106	#14 OR #15 OR #16 OR #17 OR #18 OR #19 OR #20 OR #21 OR #22 OR #23 OR #24 OR #25 OR #26 OR #27 OR #28 OR #29 OR #30 OR #31 OR #32 OR #33 OR #34 OR #35 OR #36 OR #37 OR #38 OR #39 OR #40 OR #41 OR #42 OR #43 OR #44 OR #45 OR #46	5 214 728
107	#14 OR #15 OR #16 OR #17 OR #18 OR #19 OR #20 OR #21 OR #22 OR #23 OR #24 OR #25 OR #26 OR #27 OR #28 OR #29 OR #30 OR #31 OR #32 OR #33 OR #34 OR #35 OR #36 OR #37 OR #38 OR #39 OR #40 OR #41 OR #42 OR #43 OR #44 OR #45 OR #46 OR #101	5 270 411
108	#47 OR #48 OR #49 OR #50 OR #51 OR #52 OR #53 OR #54 OR #55 OR #56 OR #57 OR #58 OR #59 OR #60 OR #61 OR #62 OR #63 OR #64 OR #65 OR #66 OR #67 OR #68 OR #69 OR #70 OR #71 OR #72 OR #73 OR #74 OR #75 OR #76 OR #77 OR #78 OR #79 OR #80 OR #81 OR #82 OR #83 OR #84 OR #85 OR #86 OR #87 OR #88 OR #89 OR #90 OR #91 OR #92 OR #93 OR #94 OR #95 OR #96 OR #97 OR #98 OR #99 OR #100	5 732 005
109	#103 AND #104 AND #107 AND #108	5 827
110	#103 AND #105 AND #107 AND #108	23 453
111	#109 OR #110	23 453
112	#111 AND [embase]/lim NOT ([embase]/lim AND [medline]/lim)	12 082

## Appendix 3

**Table 6:** Review timeline

Stage of the review at this time*	Started	Completed
Preliminary searches	Yes	Yes
Piloting of the study selection process	Yes	Yes
Formal screening of search results against eligibility criteria**	Yes	No
Data extraction	Yes	No
Data analysis	No	No

*Started: June 21, 2018, at Quebec City, Canada; *Anticipated completion*: December 20, 2020

**The results from the June search of Ovid MEDLINE were screened first. When the search was updated in October 2018, the June search results were subtracted from the October search results, leaving 435 additional references to screen. Also in October 2018, an EMBASE search was performed (see, Appendix 2 Table 5). There was inevitable, incomplete overlap between the results from the Ovid MEDLINE search and the EMBASE search, which includes the MEDLINE database. Altogether, we retrieved 19,491 references from MEDLINE database. To obtain just the references unique to the EMBASE search, the Ovid MEDLINE search results were subtracted from the EMBASE results way of a multi-step de-duplication process using Thompson EndNote, as described in Bramer 2016. The results of this EMBASE search yielded an additional 8020 references. We screened 164 full-text articles from the two databases (128 retained from MEDLINE and 36 from EMBASE). Finally, in June 2020, a search update was done in the two databases and 2661 additional references were found and extracted. We did this update according to Cochrane’s recommendations, i.e., using the dates of entry (rather than the dates of publication)

## Abbreviations

PRISMA-P: Preferred Reporting Items for Systematic Reviews and Meta-Analyses extension for protocols
PRISMA-ScR: Preferred Reporting Items for Systematic Reviews and Meta-Analyses extension for Scoping Reviews
PROBAST: Prediction study Risk of Bias Assessment Tool
